# Publication rates in animal research. Extent and characteristics of published and non-published animal studies followed up at two German university medical centres

**DOI:** 10.1371/journal.pone.0223758

**Published:** 2019-11-26

**Authors:** Susanne Wieschowski, Svenja Biernot, Susanne Deutsch, Silke Glage, André Bleich, René Tolba, Daniel Strech

**Affiliations:** 1 Institute for Ethics, History, and Philosophy of Medicine, Hannover Medical School, Hannover, Germany; 2 Institute for Laboratory Animal Science, Hannover Medical School, Hannover, Germany; 3 Institute for Laboratory Animal Science, RWTH Aachen University, Faculty of Medicine, Aachen, Germany; 4 QUEST Center for Transforming Biomedical Research, Berlin Institute of Health, Berlin, Germany; 5 Charité Universitätsmedizin Berlin, Berlin, Germany; University of Sorocaba, BRAZIL

## Abstract

Non-publication and publication bias in animal research is a core topic in current debates on the “reproducibility crisis” and “failure rates in clinical research”. To date, however, we lack reliable evidence on the extent of non-publication in animal research. We collected a random and stratified sample (n = 210) from all archived animal study protocols of two major German UMCs (university medical centres) and tracked their results publication. The overall publication rate was 67%. Excluding doctoral theses as results publications, the publication rate decreased to 58%. We did not find substantial differences in publication rates with regard to i) the year of animal study approval, ii) the two UMCs, iii) the animal type (rodents vs. non-rodents), iv) the scope of research (basic vs. preclinical), or v) the discipline of the applicant. Via the most reliable assessment strategy currently available, our study confirms that the non-publication of results from animal studies conducted at UMCs is relatively common. The non-publication of 33% of all animal studies is problematic for the following reasons: A) the primary legitimation of animal research, which is the intended knowledge gain for the wider scientific community, B) the waste of public resources, C) the unnecessary repetition of animal studies, and D) incomplete and potentially biased preclinical evidence for decision making on launching early human trials. Results dissemination should become a professional standard for animal research. Academic institutions and research funders should develop effective policies in this regard.

## Background

Animal research plays a crucial role in advancing our knowledge about biological processes and in developing safe and effective therapies, such as antibiotics, insulin, vaccines and organ transplantation [1, 2] [3]. To realize a broad knowledge gain for the wider scientific community and interested public, the results of animal studies need to be published via peer-reviewed journals or other accessible dissemination formats.

However, recent studies have indicated that a substantial proportion of completed animal studies do not publish their results. According to a Dutch survey conducted in 2012, animal researchers thought that approximately 50% of animal experiments are not published [4]. A tracking activity for studies reported in animal research abstracts presented at the 2008 Society of Critical Care Medicine Conference indicated that 38% had not been published by 2017 [5]. An assessment of publication bias in systematic reviews of interventions tested in animal studies of acute ischaemic stroke estimated a non-publication rate of 14% [6]. All of the abovementioned studies, however, applied assessment measures with limited reliability: i) Surveys assess only subjective opinions; ii) sampling conference abstracts as a starting point for assessing publication rates might already imply a bias towards desired outcomes; and iii) statistical and graphical methods to detect bias in meta-analyses, e.g., funnel-plots, bear several risks regarding misleading information [7].

Despite the methodological limitations of the existing assessments of publication rates, opinions regarding publication bias in animal research play an important role in current debates on, for example, the “reproducibility crisis” [8, 9] and “failure rates in clinical research” [10, 11]. A more thorough approach for assessing publication rates would consist of a follow-up of the full sample of protocols for all animal studies conducted at academic research institutions. Several of these studies exist for clinical research [12], but we are not aware of any study following-up animal studies from protocol to publication. German law requires that all animal studies be approved by a governmental body. The approved study protocols, together with amendments, are archived at the site of the regulatory body and at the respective animal research facility. Animal research facilities at German UMCs (university medical centres) provide quality assurance and training for those involved in animal research.

We aim to conduct the first study following-up a representative sample of animal studies from protocol to publication.

## Material and methods

The study protocol and amendments provide detailed information on methods and were pre-registered at https://osf.io/az7mt/. In the following, we report the key elements of our methods.

### Selection of proposals

A total of 105 proposals per study site were selected. The proposals were stratified by year (end of approval, 2007–2013) and categorization of animals (rodents/non-rodents). For 2007–2013, 15 proposals per year were randomly selected with 12 rodent and 3 non-rodent proposals since these numbers reflects German laboratory animal statistics (2000–2013, 75–88% rodents [13]). At one study site, we could not identify in advance proposals for which animal numbers had been reported to the state bodies with certainty. Thus, to avoid introducing bias, we also analysed proposals without certainty of their performance. We later used the animal numbers reported and amendment proposals to identify proposals that reliably had been conducted. If no such evidence existed, we excluded proposals, resulting in a total of 158 proposals. The full sample of 210 proposals was used for a sensitivity analysis.

### Data extraction

Our extraction matrix was developed using the ARRIVE (Animals in Research: Reporting In Vivo Experiments) guidelines, a previous empirical analysis of animal study protocols and a systematic review on guidelines for in vivo animal research [14–16]. We piloted this matrix with 10 proposals per study site to ensure consistent use of the matrix between researchers. Data extraction for the remaining proposals was performed by one researcher per study site.

### Publication search

This step was performed independently by two researchers for each of the 210 animal studies. We searched PubMed, Web of Science, Google Scholar, Google and Bing as well as the universities’ bibliographies using the names of the scientists involved, city and country, institution name, substance and/or intervention and species as search variables. The matching of proposals and publications was performed either by the proposal reference number or, if no reference number was cited in the publication, by applying the PICO (P: population or problem of interest; I: intervention under investigation; C: comparison of interest; O: outcomes considered most important in assessing results) scheme. After the initial publication search, the two researchers compared their results and resolved any discrepancies through discussion.

## Results

### Demographic data

We followed-up the results publication from 210 approved protocols for animal research projects whose authorization ended between 2007 and 2013. For 158 protocols, we could be certain that the experiments were completed. Of these 158 studies, 127 (80%) included rodents, and 31 (20%) included non-rodents (65% pigs, 19% sheep, 10% rabbits, 16% others such as cats, cattle, amphibia). The scope of the research as specified in the German Animal Welfare Law (§ 7a Section 1 TierSchG) was classified in the protocols as follows: 51% basic research (n = 80), 30% preclinical research (n = 48) and 18% both (n = 28). The remaining two proposals were classified as safety/preclinical and safety/basic.

### Publication rates

The overall publication rate was 67%. That is, for 106 of the 158 animal studies, we found at least one results publication (journal articles or doctoral theses) for at least one experiment that formed part of the approved protocol. Excluding doctoral theses as results publications, the publication rate decreased to 58%. For studies with publication, the number of publications per protocol ranged from 1 to 5, with a mean of 1.4. The mean time from the end of the study approval to publication was 209 days (median 169). Interrater reliability for the publication search was 78%, i.e., in 78% of all 210 follow-up cases, the raters identified the same publications initially. All other cases were later resolved through discussion (final interrater agreement = 100%). Raw data are provided in S1 Table.

Stratification by year (end of authorization) showed no clear trend for an increase or decrease in publication rates over time (see Table 1). There was also no substantial difference (>10%) in publication with regard to i) the two research sites, ii) the animal type (rodents vs. non-rodents), iii) the scope of research (basic vs. preclinical), or iv) the discipline of the applicant (physicians/dentists vs. life scientists); see Table 1 for detailed information. Comparing proposals by sample size (approved number of animals, n<207 vs. n>207), we found that larger studies were published more often than smaller studies (73% vs. 61%). We chose n = 207 because this number was the median for the number of animals across all 158 protocols.

**Table 1:** Publication rates for the full sample.

	Total number of protocols	Percentage of protocols with at least one results publication for at least one experiment
**Authorization of protocol ended (year)**		
2007	21	62%
2008	17	59%
2009	22	68%
2010	24	63%
2011	21	81%
2012	25	56%
2013	28	79%
Total	158	67%
**Study site**		
Study site 1	53	68%
Study site 2	105	67%
**Animal type**		
Rodent	127	68%
Non-rodent	31	65%
**Scope of research**		
Preclinical	48	65%
Basic	80	68%
Preclinical + basic	28	71%
Other	2	50%
**Approved number of animals**^1^		
≤ 207	79	61%
> 207	79	73%
**Acute vs. chronic projects**		
Acute	20	60%
Acute and chronic	6	67%
chronic	132	68%
**Discipline of the applicant**^**2**^		
Physicians/dentists	121	65%
Life scientists	39	69%
Veterinarians	2	100%

1 We chose n = 207 animals as the threshold because this number was the median for the number of animals across all 158 protocols.

2 The sum exceeds 158 since proposals can have more than one applicant and applicants can have more than one discipline.

As mentioned in the Methods section, we excluded from the analysis those proposals for which their performance was uncertain (n = 52). These excluded proposals had a substantially lower publication rate (46%), indicating that some of these studies were indeed not conducted.

## Discussion

This study is the first to assess the rate of results publications in animal research via a follow-up of archived animal study protocols. From the 210 studies conducted at one of two German UMCs, we identified 158 as certainly having been completed. The publication rate for the certainly completed studies was 67%. Excluding theses archived at the respective university libraries, the publication rate decreased to 58%.

In this study, we did not assess why the other 33% of animal studies did not disseminate results. We address this question in an ongoing study. We therefore do not know, for example, whether non-published studies more often had inconclusive results, involved intellectual property issues, or differed in any other way from published results.

However, the non-publication of results from completed animal studies poses several ethical and practical challenges, regardless of the underlying reasons. If animal studies that were approved because of their potential for knowledge gain do not disseminate their results even years after their completion, then their preconditioned knowledge gain for a wider scientific community is highly questionable. In addition to this foundational ethical challenge, the non-publication of results from animal studies poses several further challenges. First, publicly funded animal studies that do not disseminate their knowledge gain waste public resources. Second, if some research questions have already been studied in animals but the results were not reported to the scientific community, then other researchers might conduct the same or very similar studies with further animals or they might include experimental groups that would not be needed in light of previous studies. Non-publication thus contradicts the 3R principle of reduction and further challenges the refinement principles, which could be realized by advancing experimental planning through the integration of previous findings. Third, increasing evidence shows that published results are biased towards study results showing the desired outcome direction. For example, Emily Sena et al. showed that publication bias in reports of animal stroke studies leads to a major overstatement of efficacy [6]. In recent years, several experts, especially from the pharmaceutical industry, have expressed their concerns that this publication bias might contribute to the high failure rate of clinical trials [10, 11]. In light of the major impact of publication bias in animal research, all involved stakeholders should be aware of those implications and should make efforts to antagonize them.How can we improve publication rates in animal research, and what are the related barriers? Properly addressing these questions falls beyond the scope of this paper. In the following, we highlight some core issues that need further discussion and activities by the relevant stakeholders that fund, regulate, host, or otherwise have an impact on animal research. First, results dissemination should become a professional standard for animal research. International guidelines and laws for research with humans, such as the Declaration of Helsinki, already include results dissemination as a core requirement. More recently, internationally leading funders of biomedical research, such as the Wellcome Trust or the Horizon 2020 programme of the European Commission, have published new policies for clinical research, mandating results dissemination as a new requirement for funding [17, 18]. In addition to policy makers and funders, academic institutions must do their part as well [9]. With regard to informing early clinical trials, the challenges go beyond complete results publications in scientific journals as we pointed out in a previous work [19]. Most investigator brochures for phase I or II clinical trials primarily cite internal reports of preclinical studies as evidence base for starting a clinical trial. Preregistration of preclinical studies could provide a helpful tool to prevent selective outcome reporting and facilitate a more balanced decision making [20].

Several dysfunctional incentives for non-publication might erect one important roadblock for the unbiased results publication of animal (and clinical) research. Academic institutions, for example, reward researchers and appoint professorships mostly for publications in journals with high impact factors and for the extent of secured third-party grants. However, it is difficult to publish the results of animal studies that are inconclusive or that did not confirm the hypothesis (“negative studies”) in journals with high impact factors. Even worse, publications in journals with low impact factors reduce a researcher’s average impact factor, which is one reason why some researchers might even actively refrain from publishing inconclusive or “negative” results [21]. From the technical perspective, however, the publication of inconclusive or “negative” results is no longer a challenge. There are many journals that explicitly publish all studies, regardless of the direction of their results. In addition to standard peer-reviewed journals, new tools for results dissemination exist via preprint servers (e.g., bioRxiv) or repositories (e.g., OSF (Open Science Framework)).

Further research should validate and contextualize our results. Other national and international animal research facilities and regulatory authorities should assess the publication rates for representative samples of completed animal studies. For countries that do not have centralized archives of animal study protocols, such tracking efforts are more difficult to realize. Recent developments such as the launch of animal study registries might improve transparency by providing more fine-grained information on animal studies, which, in turn, will allow more effective and efficient tracking activities, as they are common in clinical research (www.animalstudyregistry.org or www.preclinicaltrials.eu). Baseline data for reporting rates are also important for monitoring whether reporting in animal research improves and whether new policies such as animal study registries or new institutional and funding policies at least correlate with such improvements. Further research is also needed to better understand the reasons and potential barriers behind the non-reporting of results and whether non-reported results differ significantly and substantially from reported results. Finally, analyses on the design (PREPARE (Planning Research and Experimental Procedures on Animals: Recommendations for Excellence) guidelines), reporting quality (e.g., adherence to the ARRIVE guidelines) and consistency between the statistical analyses described in the protocol and in the publication (“p-hacking”) are important to complement the picture of reporting practices in animal research. In summary, more evidence generated via meta-research on reporting practices will facilitate an important, practice-oriented discussion on how to increase value and reduce waste in animal research.

## Supporting information

S1 Table Raw data. Raw data of included proposals, cut for data that might allow identification of involved persons.
(XLSX)
